# Assessing the presence and motivations of orthorexia nervosa among athletes and adults with eating disorders: a cross-sectional study

**DOI:** 10.1007/s40519-023-01631-7

**Published:** 2023-12-09

**Authors:** Mandy Foyster, Nessmah Sultan, Matilda Tonkovic, Andrew Govus, Helen Burton-Murray, Caroline J. Tuck, Jessica R. Biesiekierski

**Affiliations:** 1 Department of Dietetics, Nutrition and Sport, La Trobe University, Bundoora, VIC Australia; 2 Department of Nutrition, Dietetics and Food, Monash University, Notting Hill, VIC Australia; 3 Division of Gastroenterology, Massachusetts General Hospital, Boston, MA USA; 4 Harvard Medical School, Boston, MA USA; 5 Department of Nursing and Allied Health, Swinburne University, Hawthorn, VIC Australia

**Keywords:** Disordered eating, Endurance athletes, Eating disorder history, Compulsive exercise, Weight control, Eating motivations

## Abstract

**Purpose:**

Orthorexia nervosa involves restricting diet based on quality rather than quantity. Although orthorexia is well reported in many at-risk populations, limited data addresses its presence in individuals with eating disorder history (EDs) or athletes. We aimed to identify the presence and potential drivers of orthorexia in adults with EDs and endurance athletes, compared to control subjects.

**Methods:**

Participants ≥ 18y included: people with a diagnosed eating disorder (ED as per DSM-5); endurance athletes (training/competing ≥ 5 h/week); or control subjects. Participants (n = 197) completed an online survey assessing orthorexia (eating habits questionnaire, EHQ), eating motivations (TEMS-B) and compulsive exercise (CET).

**Results:**

ED had the highest orthorexia symptom severity (92.0 ± 3.02, n = 32), followed by athletes (76.2 ± 2.74, n = 54) and controls (71.0 ± 1.80, n = 111) (*F* (2) = 18.2, *p* < 0.001). A strong positive correlation existed between weight control motives and higher orthorexia symptom severity (*r* = 0.54, 95% CI [1.35, 2.36], p < 0.001), while a weak negative association existed between Hunger and Pleasure motives and higher orthorexia symptom severity (*r* = 0.23, 95% CI [− 2.24, − 0.34], p = 0.008; *r* = 0.26, 95% CI [− 2.11, − 0.47], p = 0.002, respectively). A moderate positive relationship was found between CET and orthorexia symptom severity (95% CI [1.52, 3.12], p < 0.001).

**Conclusion:**

Adults with ED history and endurance athletes have greater orthorexia symptom severity compared to control. Clinicians working with at-risk populations should screen patients and be aware of red-flags of orthorexic traits, desire to control weight, and compulsive exercise behavior.

**Level of evidence:**

III: Evidence obtained from cohort studies.

**Supplementary Information:**

The online version contains supplementary material available at 10.1007/s40519-023-01631-7.

## Introduction

Orthorexia nervosa (ON) describes a pathological fixation with food based on its proposed health benefits [1]. Driven by the pursuit of optimum health, individuals with orthorexia develop self-imposed, extremely restrictive dietary rules, often associated with malnutrition, reduced quality of life, social isolation, depression, anxiety and increased suicidality [2–4]. Orthorexia is currently not recognized as a stand-alone eating disorder by the *Diagnostic and Statistical Manual of Mental Disorders* (*DSM-5*; [5]) or the International Classification of Diseases (ICD-11; [6]), and the relationship between orthorexia and other eating disorders (ED) is not well understood. Despite growing literature supporting the inclusion of orthorexia into revisions of the *DSM* [7, 8], orthorexia research is limited by poor study designs, small sample sizes, inaccurate measurement tools, and a lack of consensus on the definition and diagnostic criteria [1, 9, 10].

Prevalence rates of orthorexia have been reported to be anywhere between 0 and 97% [2, 11], in certain high-risk populations, including adults who follow restrictive eating patterns (e.g. gluten-free, dairy-free, low FODMAP[11]); adults with disorders of gut-brain interaction [11–13]; yoga practitioners (86%) [14, 15]; and Dietitians/Nutritionists (50–74%) [16–18]. Other groups considered to be at high-risk of orthorexia, including athletes and those with a history of EDs, have not been studied to date [2, 19]. Athletes, specifically those who participate in endurance (e.g. running, cycling, triathlon), aesthetic (e.g. gymnastics, synchronized swimming) and weight-class sports (e.g. boxing, martial arts, weightlifting) have a higher risk of developing an ED than athletes from other sporting styles/ less active individuals [20, 21] and may therefore be at heightened risk of orthorexia.

Factors that motivate and drive eating choices are closely linked with orthorexic behaviors, yet to date no research has certain high-risk populations. Choices around eating are often driven by various factors including price, taste, health, cultural values, habits and emotional regulation [22]. Understanding the relationship between eating motivations and orthorexia may provide valuable insight into the development of this condition and allow for early intervention, before an obsession with food manifests further along the spectrum of eating to a diagnosable eating disorder. Additionally, it has been suggested that orthorexia differs from other diagnosable EDs in its notable absence of weight loss goals [4, 7, 23], though some research suggests there may still be a preoccupation with body weight and shape and body dissatisfaction [2, 24–27]. Furthermore, the factors contributing to orthorexia in athletes/active individuals and those with a history of ED is currently understudied [20, 21, 28].

Given that orthorexia describes the pursuit of optimal health, it is logical that this pursuit extends beyond nutrition to other health enhancing behaviors including exercise. Orthorexia has been linked with perfectionistic characteristics, which may be applied to both food and exercise [28–30]. Compulsive exercise describes the rigid and highly driven urge to be physically active and an inability to stop even in light of negative consequences (injury, disrupted personal relationships, missing social events) [20, 31, 32]. The relationship between compulsive exercise and eating disorders is well established [31–34], with athletes and active individuals at higher risk of developing exercise addiction and ED’s compared to the general population [20]. However, there is limited research assessing the relationship between exercise and orthorexia [29, 35].

The primary aim of this study was to determine the presence of orthorexia among high-risk populations (people with history of an ED, and endurance athletes) compared to the control subjects. We hypothesized that those with an ED history would display the highest orthorexic symptoms, followed by athletes and control subjects. The secondary aims were to determine the relationship between orthorexia and: (1) compulsive exercise; and (2) motivational influences on food choice.

## Methods

### Study participants

Participants were recruited internationally, primarily through social media (including Instagram, Twitter and Facebook) and invited to complete an online questionnaire. Eligible participants were also recruited via clinicians including sports physicians, Australian national sporting bodies and other professional societies, including not for profit eating disorder foundations. The online survey (REDCap platform; [36]), was accessible from June 2020–August 2021 with two main recruitment periods (June–September 2020 and May–August 2021), however eating motivations and compulsive exercise questionnaires were only completed by participants in 2021.

Eligible participants were English speakers (aged ≥ 18 years), who fit one of the following categories: (a) people with a diagnosed eating disorder (active within the past 5 years (new diagnosis or relapse) and considered ‘mild’ or ‘moderate’ as per *DSM-5* criteria [5, 37] with formal diagnosis confirmed through letter from a member of the participants’ treating team (e.g. Psychologist, Doctor, Dietitian); (b) endurance athletes (defined as those who participate in endurance training/sport (running, swimming, cycling, triathlon or race walking) for ≥ 5 h per week at an international, national, or recreational level), confirmed through access to training log; or (c) control subjects, who self-reported absence of chronic health conditions (including gastrointestinal, inflammatory, immune or psychiatric) and were weight stable (within 5%) over previous three months.

**Fig. 1:**
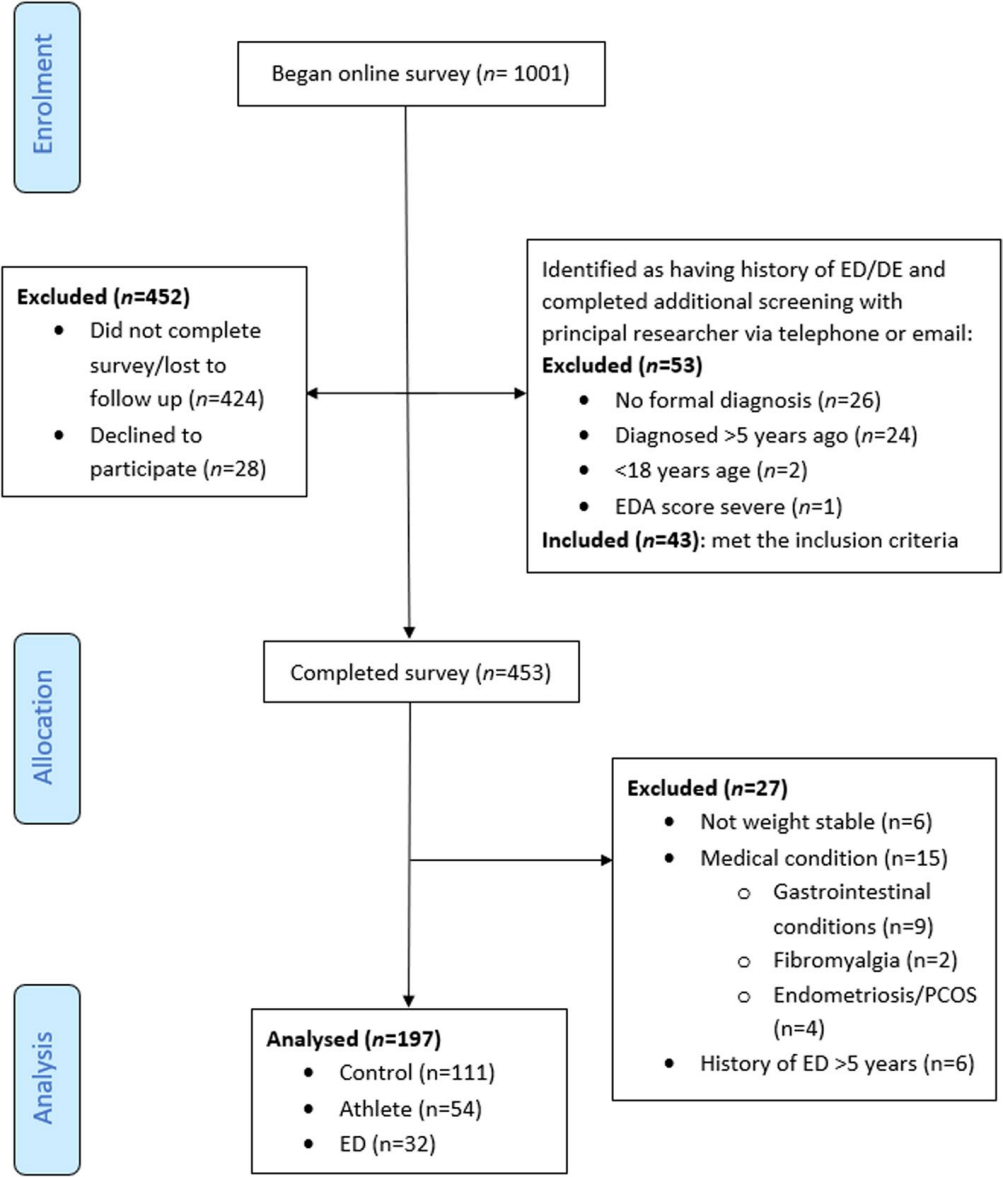
Participant selection flow chart

Upon commencement of the online survey, an initial screening questionnaire was completed, whereby participants who indicated they had a history of an ED were stopped and contact details provided to the research team. The primary researchers (Accredited Practicing Dietitians MF or NS) then contacted these participants by telephone or email for further screening via the EDA-5 questionnaire [37], to assess current level of ED symptom severity. All other participants continued the survey uninterrupted (Fig. 1). Where participants met the criteria for more than one cohort, priority was ceded to the ED cohort given the overarching nature of these illnesses, with the participant analyzed as part of the ED cohort. For example, any participant who met the athlete criteria and had an occurrence of ED within the past 5 years were classified as ED participants, and any participant who met the athlete criteria and had a history of ED with no reoccurrences within the last 5 years were included in the athlete group. Convenience sampling was used and we aimed to match the number of controls and ED to athlete participants, and a 2-year recruitment period was set. This study was approved by La Trobe University Human Research Ethics Committee on March 12th 2020 (approval number HEC20070).

### Measures

#### Demographic

Participants self-reported age, gender, location, level of education, employment status, ethnicity, past medical history, body weight, height and level of physical activity.

#### Orthorexia nervosa and disordered eating

Orthorexia was measured using the Eating Habits Questionnaire (EHQ; [38]), a 35-item questionnaire that assesses orthorexia symptomatology. The EHQ measures: (a) knowledge of healthy eating (9 items, e.g. ‘My diet is better than other people’s diets’), (b) problems associated with healthy eating (20 items e.g. ‘I turn down social offers that involve eating unhealthy food’) and (c) feeling positively about healthy eating (6 items, e.g. ‘I feel in control when I eat healthy’); hereon after referred to as ‘knowledge’, ‘problems’ and ‘feelings’ [38]. A higher overall score indicates greater orthorexia symptom severity. There is no specific cut-off score used to diagnose orthorexia [9].

Disordered eating was measured using the SCOFF (‘Sick, Control, One Stone, Fat, Food’), a 5-item survey often used as a screening tool for EDs and disordered eating [39–41]. The SCOFF comprises binary ‘yes’, ‘no’ questions with SCOFF ≥ 2 indicating possible ED [40, 42].

#### Eating motivations

The Eating Motivations Survey-Brief (TEMS-B) is a 45-item questionnaire that measures fifteen distinct motivations behind food choices, encompassing various motives including social aspects of eating, coping with negative affect, pleasure seeking, and complying with societal expectations [22]. Six motives based on the current working definition for orthorexia were chosen for this study (3-items each), four of which were highly pertinent to orthorexia: ‘health’ (e.g. ‘because it is healthy’), ‘natural concerns’ (e.g. ‘because it is organic’), ‘weight control’ (e.g. ‘because it is low in calories’) and ‘social image’ (e.g. ‘because it makes me look good in front of others’); and two antithetical motives: ‘pleasure’ (e.g. ‘because I enjoy it’) and ‘hunger and need’ (e.g. ‘because I am hungry’). TEMS-B uses a 7-point Likert scale ranging from 1 (never) to 7 (always), with scores ranging from 3–21; higher scores indicate a stronger influence on food choices.

#### Compulsive exercise

The ‘Compulsive Exercise Test’ (CET; [31]) is a 24-item questionnaire measuring compulsive exercise across 5 subscales: avoidance and rule-driven behavior (8-items e.g. ‘I feel extremely guilty if I miss an exercise session’); weight control exercise (5-items e.g. ‘I exercise to improve my appearance’); mood improvement (5-items e.g. ‘I feel less anxious after I exercise’); lack of exercise enjoyment (3-items e.g. ‘I find exercise a chore’); and exercise rigidity (3 items e.g. ‘My weekly pattern of exercise is repetitive’). Using a Likert scale from 0 (never) to 5 (always), total scores range from 0–120 with higher scores indicating more disordered exercise behaviors. No specified cut-off score has been set to identify exercise addiction.

### Statistical analysis

Data were analyzed via Jamovi 2 (The Jamovi Project [43]) after being exported from REDCap and manually cleaned using Microsoft Excel. Only complete datasets were analyzed, meaning those from participants who had completed 100% of the survey. The assumption of normality of model residuals was assessed through Shapiro Wilk tests and visual inspection of QQ plots. Equality of variance was assessed using Levene’s test and when violated, a 20% trimmed mean was used. Descriptive statistics were reported as means ± standard deviations (parametric) and medians and interquartile ranges (non-parametric). Categorical data was reported as *n* and frequencies. The normality of model residuals was checked by visual inspection of QQ plot.

For the primary aim (to assess the presence of orthorexia), a one-way Analysis of Variance (ANOVA, visually normal) or Robust ANOVA (if normality violated) with a 20% trimmed mean were used. Post-hoc, Tukey (equal between group variances) or Games-Howell (unequal between group variances) analysis examined pairwise differences between each cohort. Chi squared test was used to assess the presence of disordered eating (SCOFF). Similarly, secondary aims were analyzed using Robust ANOVA (TEMS) and one-way ANOVA (CET).

Secondary analysis included binomial logistical regression (SCOFF) and bivariate linear regression (EHQ). For both models were built sequentially with lower order effects (continuous variable and cohort) added first followed by the higher order interaction term (e.g. CET x cohort). Model parsimony was compared using Schwartz’s Bayesian Information Criteria (BIC) [44], with a lower BIC indicating a more parsimonious (simpler) model fit compared to other candidate models. CET and TEMS subscales were mean centered for analysis to avoid multicollinearity which could inflate the standard errors. A model controlling for demographic data (e.g., gender) was run, though did not improve model fit (i.e., higher BIC compared to a simpler model with less covariates) and was therefore not used in the final analysis. The probability of rating SCOFF ≥ 2 with a one unit change from the continuous variable (CET, TEMS) mean was calculated for cohort, with a 95% confidence interval denoting the imprecision of the parameter estimate.

Additionally, a bivariate linear regression was fit to examine whether the relationship between CET or TEMS subscales (continuous variables) and EHQ (continuous variable) existed while controlling for cohort and sex (excluding ED group). Models were built sequentially and parsimony compared using BIC. The probability of rating higher on the EHQ (indicating more orthorexic behaviors) with a one unit change from the continuous variable mean calculated for cohort, with a 95% confidence interval denoting the imprecision of the estimate. Pearson’s Product Moment (PPM) correlation was calculated to estimate the standardized effect size of the linear relationship between two variables. The magnitude of PPM was interpreted as: 0.1 = small, 0.3 = medium, > 0.5 = large [45]. Spearman’s rank correlation was used to assess agreement between the two disordered eating measures.

## Results

### Descriptive analysis

#### Demographic data

One thousand and one participants provided implied consent and began the survey (recruitment flow shown in Fig. 1). The final analyzed sample included 197 participants (athletes (*n* = 54), eating disorder (*n* = 32) and controls (*n* = 111), with 80.2% participants identifying as female and aged between 18–80 years old.

Demographic data for all participants is presented in Table 1. Across all cohorts, most participants identified as female (control: 84%, athlete: 61%, ED: 100%). The trimmed mean age (years) (*M:* 33.60, *SE*: 1.06) varied across cohorts (*M*: 18.9, *p* < 0.001) with differences between the athlete and ED (*F* = 9.51, *p* < 0.001), and control and ED (*F* = 10.67*, p* < 0.001). The trimmed mean BMI (kg/m^2^) also varied between cohorts (*F* = 4.69, *p* = 0.013) with variation between athlete and control (*F* = -1.37, *p* = 0.039), and control and ED (*F* = 2.05, *p* = 0.027). Most participants across groups had completed a Bachelor’s degree equivalent or above (control: 74%, athlete: 83%, ED: 69%), which did not vary between cohorts (χ^2^ (2) = 2.52, *p* = 0.284). Most participants were born (control: 55%, athlete: 75%, ED: 56%) and reside (control: 69%, athlete: 85%, ED: 62%) in Australia or New Zealand, and within metropolitan areas (control: 64%, athlete: 79%, ED: 69%).

**Table 1:** Participant characteristics by cohort

	Control (*n* = *110*)	Athlete (*n* = 54)	ED (*n* = *32*)	*p* value
Sex (n, %)				
Female	93 (85)	33 (61)	32 (100)	^a^ < 0.001*
Male	17 (15)	21 (39)	0	
Age, years, trimmed mean (range)	38.60 (19–80)	(n = 53)35.20 (18–66)	25.70 (19–52)	^b^ < 0.001*
Height, cm, trimmed mean (SE)	167 (9.24)	171 (14.02)	165 (11.57)	^b^0.007*
Body mass, kg, trimmed mean (SE)	64.95 (1.40)	64.80 (1.54)	58.0 (1.71)	^b^0.004*
BMI, kg/m^2^, trimmed mean (SE)	23.13 (0.42)	21.70 (0.42)	21.00 (0.63)	^b^0.013*
Education level, n (%)				
Bachelor’s degree and above	81 (74)	45 (83)	22 (69)	^a^0.284
Other	29 (26)	9 (16)	9 (31)	
Work status, n (%)				
Casual	7 (6)	7 (13)	9 (28)	
Part time	25 (23)	9 (17)	6 (19)	
Full time	52 (47)	36 (66)	12 (37)	^a^ < 0.001*
Retired	13 (12)	2 (4)	0	
Unemployed	13 (12)	0	5 (16)	
Region of birth, n (%)				
Africa	2 (2)	0	0	
Asia	8 (7)	1 (2)	1 (3)	
Australia/NZ	60 (54)	41 (76)	18 (57)	
Europe	26 (24)	6 (11)	10 (31)	^a^0.156
North America	11 (10)	6 (11)	3 (9)	
South America	3 (3)	0	0	
Region of residence, n (%)				
Asia	2 (2)	0	1 (3)	
Australia/NZ	76 (69)	46 (85)	20 (63)	
Europe	21 (19)	4 (7)	7 (22)	^a^0.005*
North America	10 (9)	4 (7)	4 (12)	
South America	1 (1)	0	0	
Ethnicity, n (%)	(n = 109)	(n = 53)		
Asian	10 (9)	0	0	
Australian/NZ	60 (55)	43 (81)	18 (56)	
European	28 (26)	7 (13)	12 (38)	^a^0.017*
North American	8 (7)	2 (4)	2 (6)	
South American	3 (3)	1 (2)	0	
Location, n (%)				
Metropolitan	70 (64)	43 (80)	22 (69)	^a^0.166
Regional	28 (25)	11 (20)	10 (31)	
Rural	12 (11)	0	0	

*ED* eating disorder, *BMI* body mass index, *SE* standard error, *NZ* New Zealand

^†^Gastrointestinal specific alternative medicines includes probiotics

^a^Chi squared test

^b^Robust ANOVA with a 20% trimmed mean

^*^Statistically significant result, p < 0.05

#### Orthorexia symptom severity and disordered eating

The ED cohort displayed the highest orthorexia symptom severity (EHQ *M:* 92.0, *SE:* 3.02; SCOFF *M:* 2.34 ± 1.31), with 75% scoring ≥ 2 on the SCOFF (Table 2), compared to athletes (EHQ *M*:76.2, *SE:* 2.74; SCOFF* M* 1.21 ± 1.33) and control (EHQ *M*: 71.0, *SE*: 1.80; SCOFF *M*: 1.07 ± 1.30).

**Table 2:** Orthorexic traits across cohorts

	Control (*n* = *110*)	Athlete (*n* = 54)	ED (*n* = *32*)	*p* value
*Eating habits questionnaire*				
Total, trimmed mean (SE)	71.0 (1.80)	76.2 (2.74)	92.0 (3.02)	^a^ < 0.001*
Knowledge	21.1 (0.68)	19.4 (0.79)	24.4 (0.66)	^b^0.009*
Feelings	18.1 (1.26)	13.3 (0.43)	14.8 (0.60)	^b^ < 0.001*
Problem	28.9 (1.74)	43.5 (1.56)	52.5 (2.18)	^a^ < 0.001*
*SCOFF*				
Total score, n (%)				
0	53 (48)	23 (43)	4 (13)	
1	22 (20)	9 (17)	4 (13)	
2	18 (17)	14 (26)	8 (25)	^c^0.001*
3	9 (8)	4 (7)	9 (28)	
4	7 (6)	3 (5)	7 (21)	
5	1 (1)	1 (2)	0	
SCOFF total ≥ 2, n (%)	35 (32)	20 (37)	24 (75)	^c^ < 0.001*

*ED* eating disorder, *SCOFF* Sick, Control, One-Stone, Fat, Food

^a^Robust ANOVA, 20% timed mean with post hoc analysis

^b^One-Way ANOVA with post hoc Tukey test

^c^Chi squared test *statistically significant, *p* < 0.05

A robust ANOVA with 20% trimmed mean showed differences between cohorts for EHQ total score (*F* (2) = 18.2, *p* < 0.001) and subscale ‘Problems’ (*F* (2) = 37.9, *p* < 0.001), with a one-way ANOVA showing between cohort differences for subscales ‘Knowledge’ (*F* (2) = 4.85, *p* = 0.009) and ‘Feelings’ (*F* (2) = 13.73, *p* < 0.001). Bivariate linear regression for the EHQ total score showed differences between ED and control (*t* (2) = 16.74, *p* < 0.001) and athlete and ED cohorts (*t* (2) = -13.6, *p* < 0.001), with nil differences between athlete and control found (*t* (2) = 3.13, *p* = 0.279; Table 3).

**Table 3:** Bivariate linear regression of EHQ total and subscales ‘Knowledge’, ‘Feelings’ and ‘Problems’ across cohorts

*Model parameters*	EHQ total			Knowledge subscale			Feelings subscale			Problem subscale		
	*Est*	*95% CI*	*p-value*	*Est*	*95% CI*	*p-value*	*Est*	*95% CI*	*p-value*	*Est*	*95% CI*	*p-value*
Intercept	73.16	[69.90, 76.43]	< 0.001*	23.66	[21.60, 25.72]	< 0.001*	21.24	[19.4, 23.11]	< 0.001*	30.50	[28.09, 33.00]	< 0.001*
*COHORT*												
Athlete vs. Control	3.13	[− 2.56, 8.83]	0.279	− 1.82	[− 3.75, 0.12]	0.066	− 8.09	[− 11.4, − 4.82]	< 0.001*	13.00	[8.80, 17.30]	< 0.001*
ED vs. control	16.74	[9.86, 23.62]	< 0.001*	2.25	[− 0.09, 4.59]	0.060	− 6.55	[− 10.50, − 2.60]	0.001*	21.0	[15.92, 26.20]	< 0.001*
Athlete vs. ED	− 13.6	[− 21.3, − 5.97]	< 0.001*	− 4.06	[− 6.66, − 1.47]	0.002*	− 1.54	[− 5.93, 2.85]	0.490	− 8.01	[− 26.20, − 15.92]	0.006*

*EHQ* eating habits questionnaire, *CI* confidence interval, *Est* estimate, *ED* eating disorder

^*^Statistically significant result, p < 0.05

A chi squared test showed differences between cohorts for SCOFF as a binary (≥ 2/ < 2), whereby scores ≥ 2 indicate risk of disordered eating (χ^2^ (2) = 19.8, *p* < 0.001). A binomial logistical regression showed differences between groups with ED 63% more likely to score ≥ 2 on SCOFF compared to athletes (95% CI [1.93, 13.48], *p* = 0.001) and 83% more likely to score ≥ 2 on SCOFF compared to controls (95% CI [0.06, 0.38], *p* < 0.001). However, no difference between the athlete and controls was found (95% CI [0.40, 1.55], *p* = 0.482) (Table 4).

**Table 4:** Binomial logistical regression of SCOFF total ≥ 2 across cohorts

Model parameters	SCOFF total ≥ 2		*95% CI*		
	Est	OR	Lower	Upper	P-value
Intercept	0.76	2.14	1.43	3.20	< 0.001*
*COHORT*					
Athlete vs. Control	− 0.23	0.79	0.40	1.57	0.506
ED vs. Control	− 1.86	0.16	0.06	0.38	< 0.001*
Athlete vs. ED	1.63	5.10	1.93	13.48	0.001*

*SCOFF* ‘Sick, Control, One-Stone, Fat, Food’,  *Est* estimate, *OR* odds ratio, *ED* eating disorder

^*^Statistically significant result, p < 0.05

**Fig. 2:**
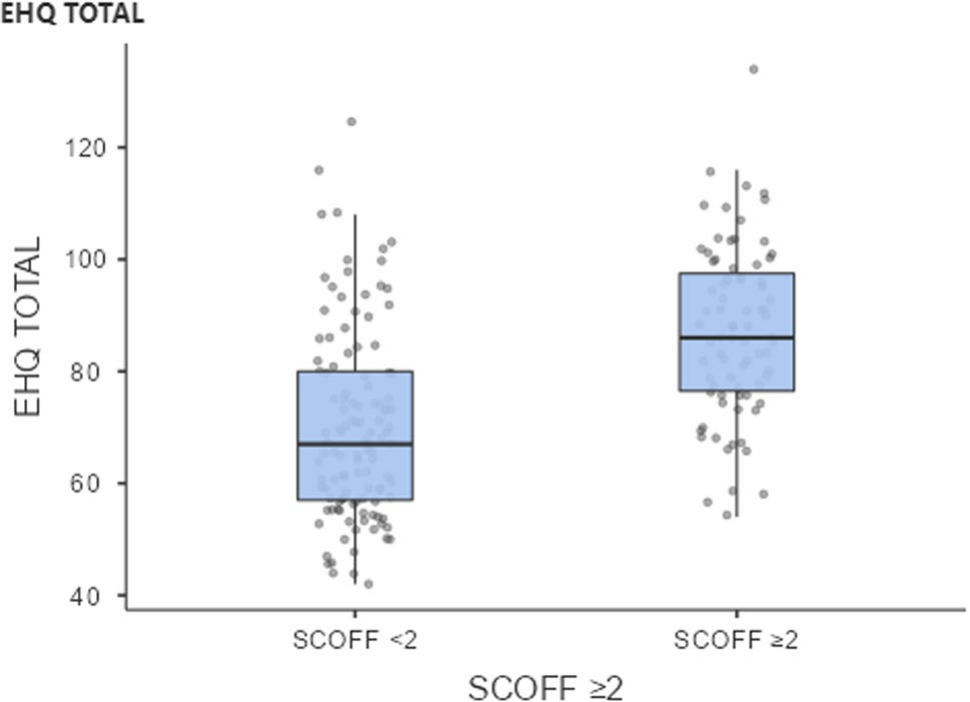
Relationship between two measures of orthorexia: EHQ and SCOFF ≥ 2, whereby ‘Yes’ indicates disordered eating (SCOFF score of ≥ 2)

Finally, a Kendall’s tau-b correlation showed a moderate positive relationship between SCOFF ≥ / < 2 and EHQ (τb = 0.399, *p* < 0.001) indicating agreement between these two measures (Fig. 2).

### Eating motivations

Lower scores were reported by the ED group for adaptive eating motivations: ‘hunger’ (*M*: 14.00 (1.03)) and ‘pleasure’ (*M*: 9.91 (1.33)), compared to athlete (*M*: 16.82 (0.39), *M*: 13.65 (0.51)) and control (*M*: 16.89 (0.41) and *M*: 12.89 (0.57)) respectively (Table 5). On average, the ED group rated 2.12 (95% CI [0.18, 4.06], *t* (127) = 2.29, *p* = 0.06) units lower on the ‘pleasure’ subscale compared to control and 2.87 (95% CI [0.93, 4.81], *t* (127) = 3.07, *p* = 0.007) units lower compared to athletes. On the ‘hunger’ subscale, the ED cohort on average rated 2.43 (95% CI [0.72, 4.14], *t* (127), *p* = 0.008) units lower than control and 2.77 (95% CI [1.06, 4.48], *t* (127) = 3.43, *p* = 0.002) units lower compared to athletes.

**Table 5:** Mean ± standard deviation responses for TEMS subscales by cohort

TEMS Subscale, Trimmed mean (SE)	Control (*n* = 59)	Athlete (*n* = 54)	ED (*n* = 17)	*p* value
Hunger	16.89 (0.41)	16.82 (0.39)	14.00 (1.03)	^a^0.033*
Pleasure	12.89 (0.57)	13.65 (0.51)	9.91 (1.33)	^a^0.034*
Health	15.95 (0.55)	16.29 (0.51)	15.91 (1.34)	^a^0.884
Natural concerns	9.16 (0.96)	9.59 (0.80)	9.18 (1.96)	^a^0.934
Weight control	7.32 (0.78)	10.29 (1.10)	11.82 (1.29)	^a^0.007*
Social image	4.51 (0.37)	4.71 (0.55)	4.91 (1.20)	^a^0.919

*TEMS* The Eating Motivations Survey, *SE* standard error, *ED* Eating disorder

^a^Robust ANOVA with a 20% trimmed mean

^*^statistically significant result, p < 0.05

Higher scores were reported by the ED group for ‘weight control’ (*M*: 11.82 (1.29)) compared to other groups (athlete *M*: 10.29 (1.10); control *M*: 7.32 (0.78)). The ED cohort on average rated 3.90 (95% CI [1.99, 5.81], *t* (127) = 3.00, *p* = 0.009) units higher than controls with no difference found between ED and athlete cohorts (95% CI [-0.46, 3.36], *t* (127) = 1.10, *p* = 0.513). No difference was found between cohorts for ‘health’, ‘natural concerns’ or ‘social image’.

A one unit change in ‘hunger’ was associated with negligible change in SCOFF (95% CI [0.99, 1.30], *p* = 0.060) and ~ 1 unit decrease in EHQ (*r* = 0.231, 95% CI [-2.24, -0.34], *p* = 0.008) (Supplementary Tables 1, 2). Similarly, a one unit change in ‘pleasure’ was associated with a ~ 1 unit decrease in EHQ (*r* = 0.264, 95% CI [-2.11, -0.47], *p* = 0.002) though the relationship with SCOFF was not significant (95% CI [0.82, 1.04], *p* = 0.214). A one unit change in ‘weight control’ was associated with ~ 1 unit increase in SCOFF (95% CI [0.75, 0.89], *p* < 0.001) and a ~ 2 unit increase in EHQ (r = 0.54, 95% CI [1.35, 2.36]), *p* < 0.001) scores (Supplementary Tables 1, 2).

Multiple regression analysis indicated that the strongest explainers of EHQ total were ‘weight control’ (adjusted R^2^ = 0.29, F (1, 129) = 53.00 *p* < 0.001), ‘natural concerns’ (adjusted R^2^ = 0.20, F (6, 3, 127) = 12.01, *p* < 0.001) and ‘health’ (adjusted R^2^ = 0.14, F (1, 129) = 22.87 *p* < 0.001) explaining ~ 30%, ~ 20% and ~ 15% of variance respectively. ‘Hunger’, ‘pleasure’ and ‘social image’ each explained ~ 5% variance (Supplementary Table 2).

### Compulsive exercise

The ED cohort displayed the highest levels of compulsive exercise (*M*: 13.0, *IQR:* 5.0) followed by the athlete (M: 12.0, IQR: 4.0) and control group (M: 11.0, IQR: 4.0; F = 3.13, p = 0.006), with differences between all subscales found (Table 6). The athlete group on average rated 1.74 (95% CI [0.95, 2.53], *t* (127) = 2.91, p = 0.012) units higher on the CET compared to the control group, while the ED group on averaged rates 2.44 (95% CI [0.37, 4.51], t (127) = 2.79, *p* = 0.017) units higher than the control. A positive relationship was found between CET and SCOFF score, whereby a one unit change in CET was associated with a ~ 0.7 unit (95% CI [0.63, 0.83], *p* < 0.001) increase in SCOFF score (Supplementary Table 3). Similarly, a moderate positive relationship was found between CET and EHQ with a one unit increase in CET is associated with ~ 2.3 unit (95% CI [1.52, 3.12], p < 0.001) increase in EHQ total score (Supplementary Table 4).

**Table 6:** Mean ± standard deviation responses for compulsive exercise by cohort

CET Global and CET subscales, Median (IQR)	Control (*n* = 59)	Athlete (*n* = 54)	ED (*n* = 17)	p value
CET global	11.00 (4.00)	12.00 (4.00)	13.00 (5.00)	0.006*
Rule driven	1.00 (2.00)	2.00 (2.00)	2.00 (3.00)	< 0.001*
Weight control	2.00 (1.00)	2.00 (2.00)	3.00 (1.00)	0.036*
Mood enhancement	3.00 (2.00)	4.00 (1.00)	3.00 (2.00)	0.006*
Lack of joy	1.00 (1.00)	1.00 (1.00)	2.00 (2.00)	< 0.001*
Exercise rigidity	2.00 (1.50)	3.00 (2.00)	3.00 (1.00)	0.027*

*ED* eating disorder, *IQR* interquartile range, *CET* compulsive exercise test

Statistical analysis completed using one-way ANOVA (Welch’s) with Tukey post-hoc analysis

^*^Statistically significant result, p < 0.05

## Discussion

The present study aimed to assess the presence of orthorexia and disordered eating among three cohorts: people with a history of an eating disorder (ED), endurance athletes and the general population (control group). As hypothesized, the orthorexia symptom severity was highest among people with a history of an ED, followed by athletes, then control subjects. A positive linear relationship was observed between compulsive exercise habits and both orthorexia and disordered eating. While other studies have explored eating motivations and orthorexia [46], this is the first study to examine this relationship using The Eating Motivations Survey. Orthorexia was negatively associated with adaptive eating motivations including ‘hunger’ and ‘pleasure’ and positively associated with ‘weight control’ indicating desire to manipulate weight and shape may be a strong motivator for food choices in those with orthorexia.

Data from the present study indicated differences between cohorts for EHQ total and all subscales, with the ED cohort displaying the highest orthorexic symptoms. While the EHQ is yet to be extensively used in research, our results align with those from other research groups [11, 47–49] who showed that individuals following restrictive eating patterns (e.g. those with a past/present experience of an eating disorder) displayed higher orthorexia symptom severity. Compared to the literature, all groups in the current study displayed higher than expected levels of disordered eating (ED 75%, Athlete 37%, Control 32% as measured by the SCOFF), possibly associated with convenience sampling (mostly conducted through social media).

Alternatively, our results may be related to the dominant sociocultural belief of healthism and the development of an orthorexic society [50–54]. Healthism places the pursuit of health (and prevention of ill health) at the top of the moral virtue ladder, encouraging individuals to pursue it at all costs. In turn, an orthorexic society is created whereby orthorexic behaviors are normalized (and often praised), resulting in the development of a “socially acceptable eating disorder” or pseudo recovery for people experiencing diagnosable eating disorders [10, 54–58]. In a systematic literature review on the psychosocial risk factors of orthorexia, McComb and Mills [2] reported that a history of an eating disorder is the strongest predictor of developing orthorexia, with previous engagement in dieting and poor body image also strong explainers [25, 59, 60]. This study found that higher orthorexia symptoms were associated with past eating disorder history and as such, support the association between the development of orthorexia and a history of an eating disorder, though a causal direction is yet to be established [23, 61].

We found that endurance athletes had significantly higher orthorexia symptom severity compare to control subjects, supporting previous research that found a positive association between orthorexia and participating in endurance sports [4, 35], spending longer duration training [4] and focusing on calories burned during training [28] among active individuals. This present study is the first to assess the presence of orthorexia among endurance athletes and confirms this population may be more likely to develop orthorexia symptoms.

This study found a positive relationship between compulsive exercise, orthorexia and disordered eating, though previous literature suggests the relationship between exercise and orthorexia is unclear. In a systematic review, McComb and Mills [2] found exercise to be positively associated with orthorexia among university student athletes [62, 63] and European women [64], with exercise frequency, compulsive exercise and investment in being physically fit associated with higher levels of orthorexia [3, 64, 65]. However, this review also found that exercising for internal motivation, health improvement and psychological improvement were not associated with orthorexia[3]. Interestingly, in more recent systematic review, Strahler et al. [35], found a small correlation between exercise and orthorexia, suggesting that regular exercise may impact orthorexia development, though is unlikely to be the most critical risk factor in developing orthorexia. A growing body of research suggests that orthorexia is more than an obsession with healthy eating, but rather a fixation on pursuing ‘perfect’ health at all costs, inclusive of compulsive exercise and other health enhancing behaviors such as reducing alcohol intake and cessation of smoking [28, 60, 66, 67]. People with high orthorexia symptoms may also not enjoy the ‘pleasure side’ of physical activity, but rather view activity as a tool to achieve their pursuit of optimal health [28], characteristics reminiscent of compulsive exercise. Our results support this definition of orthorexia owing to the positive correlation between orthorexia and compulsive exercise, though a causal direction is yet to be established due to our cross-sectional study design.

The present study is the first of its kind to use TEMS to assess the relationship between eating motivations and orthorexia [22]. Understanding motivations for eating can help to identify both normal or adaptive eating and pathological or maladaptive eating [68]. In a cross-sectional study of 605 college students, Rodgers [69] found that restrictive eating patterns are associated with less positive eating behaviors, even if the restriction is motivated by the premise of improved health. Our study found a negative correlation between orthorexia and two adaptive eating behavior motives, ‘pleasure’ and ‘hunger’, indicating that individuals with orthorexia are not driven by these motives when it comes to food choice and that their eating behaviors may be less intuitive. Conversely, as predicted, eating motivations considered to be core components of orthorexia, such as ‘health’, ‘natural concern’ and ‘social image’ were all positively correlated with orthorexia.

Our study found that ‘weight control’ is positively associated with orthorexia symptoms, further advancing our understanding of the relationship between orthorexia and weight. Of the six eating motivations analyzed, ‘weight control’ was the strongest explainer of orthorexia symptoms, and the only motive to show positive correlations on both the SCOFF and EHQ. To date, the relationship between orthorexia and weight is yet to be established, with some research suggesting that a lack of weight loss focus is a key distinction between orthorexia and other eating disorders [1, 4, 7]. Yet, other research indicates that a desire to manipulate weight and shape, and a drive for thinness and body dissatisfaction are core components of orthorexia [10, 24, 25, 34, 70], a theory supported by the findings of this study. Given that orthorexia is defined as the pursuit of optimal health, weight manipulation may be a component of orthorexia, as weight is commonly used as a proxy marker for health in an orthorexic society [19, 51]. There is however consensus among researchers that orthorexia does not discriminate by body size or shape and that body mass or body composition has little to do with orthorexia development [2, 71]. Furthermore, dieting has been shown to be a strong predictor of orthorexia [31, 33, 61, 72, 73], with people in larger bodies more likely to diet [74–76]. Thus, orthorexia can occur across the weight spectrum, with those in larger bodies potentially more likely to develop orthorexia, an important consideration for clinical practice.

### Strengths and limits

Several measurement tools are available to assess orthorexia, though all have been criticized for their lack of validity and sensitivity leading to an overestimation of prevalence rates [9, 55]. A strength of the present study was using two validated measures, the Eating Habits Questionnaire (EHQ) and the SCOFF (‘Sick, Control, One-Stone, Fat, Food’) to measure both orthorexia and disordered eating. This study found agreement between the SCOFF and EHQ, a finding which has not been reported in literature previously. Given that the SCOFF is a shorter 5-item questionnaire (compared to the 35-item EHQ), this may highlight the SCOFF as a preferential screening tool for orthorexia / disordered eating within the clinical setting. Further research in more diverse populations is needed to determine this.

Given the aforementioned limitations of orthorexia measurement tools, the use of two validated screening tools to assess both orthorexia (EHQ) and disordered eating (SCOFF) is a major strength of this study. All measurement tools used were reliable and validated in appropriate populations. However, there are several limitations to consider, all data gathered was based on self-reported questionnaires, collected at one-time point via convenience sampling which may limit generalisability to the broader ED and athlete populations. The study cohort was less culturally and ethnically diverse, predominately female, younger and more highly educated than the general population. The gender imbalance may limit the generalizability of this research study, though it is well known that females are more likely to contribute to research in this field [77]. This study was entirely advertised and conducted online, predominately through social media accounts, most of whom likely have followers who are more ‘health conscious’ and thus may not truly be representative of the general population [56, 78].

This study found that people with a history of an eating disorder and endurance athletes displayed greater orthorexia symptoms compared to control subjects. Additionally, it highlights that given healthism and an orthorexic society, orthorexia may be more prevalent among the society than previously expected. As such, clinicians must be aware of red flags of orthorexic behaviors, including obsessions with food, “clean”, “healthy” or “natural” eating, compulsive or excessive exercise and a focus on manipulating weight or shape. This study showed agreement between the two measures of orthorexia and disordered eating (EHQ and SCOFF), suggesting the simpler, 5-item SCOFF questionnaire may allow for quicker clinical screening. Further longitudinal study designs, with larger, more diverse populations are required to establish the causal link between orthorexia, compulsive exercise and eating motivations. Additionally, research assessing the relationship between the SCOFF and other measures of orthorexia is warranted to determine the clinical applicability of this simple 5-item questionnaire in assessing for orthorexia, and its applicability to specific population groups.

*What is already known on this subject?*

Despite the growing interest in orthorexia and its high prevalence rates in various populations, some high-risk groups, such as athletes and individuals with a history of eating disorders, have not yet been thoroughly examined. Investigating the link between eating motivations and orthorexia could offer valuable insights into the development of this condition and enable early intervention.

*What this study adds?*

This study found that orthorexia symptom severity was higher in people with a history of an eating disorder and in endurance athletes. Weight control as an eating motivation was strongly associated with orthorexia. These findings have implications for practice including that clinicians should be aware of orthorexic behaviors and consider screening for orthorexia in individuals with an eating disorder history, endurance athletes and/or active individuals, and those who have weight control/manipulation goals.

### Supplementary Information

Below is the link to the electronic supplementary material.

Supplementary file1 (DOCX 71 KB)

## Data Availability

Full data available from the corresponding author upon request.
