# Supplementary material for: Assessing the presence and motivations of orthorexia nervosa among athletes and adults with eating disorders: a cross-sectional study
Source: Eat Weight Disord. 2023 Dec 9;28(1):101. doi: 10.1007/s40519-023-01631-7 (PMC10710386; doi:10.1007/s40519-023-01631-7)
Supplement: Supplementary file 1 — Supplementary file1 (DOCX 71 KB) [file 40519_2023_1631_MOESM1_ESM.docx]

**Additional Tables**

**Table S1:** Relationship between TEMS subscales and orthorexia (SCOFF ≥2) using binomial logistical regression with modelling

|  |  | **Model 1** | | | | **Model 2** | | | | **Model 3** | | | |
| --- | --- | --- | --- | --- | --- | --- | --- | --- | --- | --- | --- | --- | --- |
|  | **Model Parameters** | **Est.** | ***OR*** | ***95% CI*** | ***p*-value** | **Est.** | **OR** | ***95% CI*** | ***p*-value** | **Est.** | **OR** | ***95% CI*** | ***p*-value** |
| **HUNGER** | Intercept | -2.13 | 0.12 | [0.02, 0.90] | 0.039* | -1.09 | 0.34 | [0.04, 3.06] | 0.333 | 0.08 | 1.08 | [0.06, 20.95] | 0.96 |
|  | HUNGER | 0.16 | 1.18 | [1.04, 1.33] | 0.010* | 0.13 | 1.14 | [0.99, 1.30] | 0.060* | 0.06 | 1.06 | [0.89, 1.27] | 0.532 |
|  | COHORT |  |  |  |  |  |  |  |  |  |  |  |  |
|  | Athlete vs. Control |  |  |  |  | -0.55 | 0.58 | [0.26, 1.30] | 0.184 | 0.01 | 1.01 | [0.75, 1.37] | 0.927 |
|  | ED vs. Control |  |  |  |  | -1.97 | 0.14 | [0.14, 0.39] | 0.508 | 1.2 | 3.31 | [0.77, 14.18] | 0.107 |
|  | Athlete vs. ED |  |  |  |  | 1.42 | 4.12 | [1.13, 15.00] | 0.032* | -1.2 | 0.3 | [0.07, 1.29] | 0.107 |
|  | McFadden's R^2^/BIC | 0.04/176 |  |  |  | 0.10/175 |  |  |  | 0.15/176 |  |  |  |
| **HEALTH** | Intercept | 0.98 | 2.66 | [0.56, 12.72] | 0.222 | 1.35 | 3.87 | [0.71, 21.19] | 0.119 | 1.29 | 3.64 | [0.38, 34.74] | 0.261 |
|  | HEALTH | -0.03 | 0.97 | [0.88, 1.07] | 0.552 | -0.02 | 0.98 | [0.88, 1.08] | 0.674 | -0.02 | 0.98 | [0.85, 1.13] | 0.8 |
|  | COHORT |  |  |  |  |  |  |  |  |  |  |  |  |
|  | Athlete vs. Control |  |  |  |  | -0.47 | 0.63 | [0.28, 1.40] | 0.254 | -0.05 | 0.95 | [0.76, 1.19] | 0.643 |
|  | ED vs. Control |  |  |  |  | -2.18 | 0.11 | [0.03, 0.40] | <0.001* | 0.15 | 1.16 | [0.81, 1.65] | 0.424 |
|  | Athlete vs. ED |  |  |  |  | 1.71 | 5.55 | [1.59, 19.39] | 0.007* | -0.2 | 0.82 | [0.57, 1.20] | 0.295 |
|  | McFadden's R^2^/BIC | 0.00/183 |  |  |  | 0.08/179 |  |  |  | 0.09/187 |  |  |  |
| **PLEASURE** | Intercept | 0.76 | 2.15 | [0.54, 8.49] | 0.277 | 1.98 | 7.27 | [1.38, 38.30] | 0.019* | 1.67 | 5.34 | [0.56, 50.75] | 0.145 |
|  | PLEASURE | -0.02 | 0.98 | [0.89, 1.09] | 0.714 | -0.07 | 0.93 | [0.82, 1.04] | 0.214 | -0.05 | 0.95 | [0.80, 1.12] | 0.546 |
|  | COHORT |  |  |  |  |  |  |  |  |  |  |  |  |
|  | Athlete vs. Control |  |  |  |  | -0.44 | 0.65 | [0.29, 1.44] | 0.286 | 0.04 | 1.04 | [0.80, 1.34] | 0.783 |
|  | ED vs. Control |  |  |  |  | -2.39 | 0.09 | [0.02, 0.34] | <0.001* | -0.63 | 0.53 | [0.19, 1.51] | 0.235 |
|  | Athlete vs. ED |  |  |  |  | 1.95 | 7.05 | [1.87, 26.65] | 0.004* | 0.67 | 1.95 | [0.66, 5.35] | 0.212 |
|  | McFadden's R^2^/BIC | 0.00/183 |  |  |  | 0.01/177 |  |  |  | 0.11/183 |  |  |  |

**Table S1** continued

|  |  | **Model 1** | | | | **Model 2** | | | | **Model 3** | | | |  |  |  |  |  |
| --- | --- | --- | --- | --- | --- | --- | --- | --- | --- | --- | --- | --- | --- | --- | --- | --- | --- | --- |
|  | **Model Parameters** | **Est.** | ***OR*** | ***95% CI*** | ***p*-value** | **Est.** | **OR** | ***95% CI*** | ***p*-value** | **Est.** | **OR** | ***95% CI*** | ***p*-value** |  |  |  |  |  |
| **NATURAL CONCERNS** | Intercept | 1.21 | 3.34 | [1.53, 7.31] | 0.003* | 1.83 | 6.26 | [2.37, 16.56] | <0.001 | 1.8 | 6.08 | [1.69, 21.79] | 0.006* |  |  |  |  |  |
|  | NATURAL CONCERNS | -0.07 | 0.93 | [0.87, 1.00] | 0.047* | -0.08 | 0.92 | [0.86, 0.99] | 0.030* | -0.08 | 0.93 | [0.83, 1.03] | 0.152 |  |  |  |  |  |
|  | COHORT |  |  |  |  |  |  |  |  |  |  |  |  |  |  |  |  |  |
|  | Athlete vs. Control |  |  |  |  | -0.48 | 0.62 | [0.27, 1.39] | 0.243 | 0.00 | 0.99 | [0.85, 1.16] | 0.951 |  |  |  |  |  |
|  | ED vs. Control |  |  |  |  | -2.3 | 0.1 | [0.03, 0.36] | <0.001* | 0.01 | 0.99 | [0.78, 1.27] | 0.963 |  |  |  |  |  |
|  | Athlete vs. ED |  |  |  |  | 1.82 | 6.17 | [1.72, 22.20] | 0.005* | 0 | 1 | [0.78, 1.28] | 0.994 |  |  |  |  |  |
|  | McFadden's R^2^/BIC | 0.02/179 |  |  |  | 0.11/174 |  |  |  | 0.11/184 |  |  |  |  |  |  |  |  |
| **WEIGHT CONTROL** | Intercept | 2.55 | 12.87 | [4.76, 34.73] | <0.001* | 2.7 | 14.81 | [5.11, 42.84] | <0.001* | 3.17 | 23.8 | [4.64, 122.15] | <0.001* |  |  |  |  |  |
|  | WEIGHT CONTROL | -0.21 | 0.81 | [0.75, 0.89] | <0.001* | -0.2 | 0.82 | [0.75, 0.90] | <0.001* | -0.25 | 0.78 | [0.67, 0.92] | 0.781 |  |  |  |  |  |
|  | COHORT |  |  |  |  |  |  |  |  |  |  |  |  |  |  |  |  |  |
|  | Athlete vs. Control |  |  |  |  | 0 | 1 | [0.41, 2.45] | 1 | 0.12 | 1.12 | [0.92, 1.37] | 0.256 |  |  |  |  |  |
|  | ED vs. Control |  |  |  |  | -1.71 | 0.18 | [0.05, 2.45] | 0.012* | -0.25 | 0.78 | [0.46, 1.33] | 0.357 |  |  |  |  |  |
|  | Athlete vs. ED |  |  |  |  | 1.71 | 5.51 | [1.45, 20.91] | 0.012* | 0.37 | 1.44 | [0.86, 2.42] | 0.17 |  |  |  |  |  |
|  | McFadden's R^2^/BIC | 0.15/157 |  |  |  | 0.20/159 |  |  |  | 0.21/165 |  |  |  |  |  |  |  |  |
| **SOCIAL IMAGE** | Intercept | 1.23 | 3.43 | [1.62, 7.25] | 0.001* | 1.66 | 5.27 | [2.16, 12.87] | <0.001* | 2.1 | 8.18 | [2.09, 32.02] | 0.003* |  |  |  |  |  |
|  | SOCIAL IMAGE | -0.13 | 0.88 | [0.78, 0.99] | 0.031* | -0.12 | 0.88 | [0.78, 1.00] | 0.054 | -0.21 | 0.81 | [0.65, 1.02] | 0.074 |  |  |  |  |  |
|  | COHORT |  |  |  |  |  |  |  |  |  |  |  |  |  |  |  |  |  |
|  | Athlete vs. Control |  |  |  |  | -0.42 | 0.66 | [0.29, 1.47] | 0.308 | 0.12 | 1.12 | [0.85, 1.50] | 0.412 |  |  |  |  |  |
|  | ED vs. Control |  |  |  |  | -2.16 | 0.12 | [0.03, 0.42] | <0.001* | 0.13 | 1.14 | [0.77, 1.69] | 0.512 |  |  |  |  |  |
|  | Athlete vs. ED |  |  |  |  | 1.73 | 5.66 | [1.59, 20.21] | 0.008* | -0.01 | 0.99 | [0.69, 1.42] | 0.944 |  |  |  |  |  |
|  | McFadden's R^2^/BIC | 0.03/178 |  |  |  | 0.10/175 |  |  |  | 0.11/185 |  |  |  |  |  |  |  |  |

Abbreviations: Est, Estimate; SE, Standard Error; OR, Odds Ratio, CI, Confidence Interval

Statistical analysis conducted by sequential logistical regression, *statistically significant result, p<0.05

**Table S2:** Relationship between The Eating Motivations Survey (TEMS) subscales and orthorexia nervosa as measured by EHQ

|  | **Model 1** | | | **Model 1** | | | **Model 1** | | |
| --- | --- | --- | --- | --- | --- | --- | --- | --- | --- |
|  | **EHQ.TOTAL** | | | **EHQ.TOTAL** | | | **EHQ.TOTAL** | | |
| *Parameters* | *Estimates* | *95% CI* | *p* | *Estimates* | *95% CI* | *p* | *Estimates* | *95% CI* | *p* |
| (Intercept) | 96.98 | [81.22, 112.75] | <0.001* | 92.43 | [81.50, 103.35] | <0.001* | 48.27 | [36.53, 60.01] | <0.001* |
| Hunger | -1.29 | [-2.24, -0.34] | 0.008* |  |  |  |  |  |  |
| Pleasure |  |  |  | -1.29 | [-2.11, -0.47] | 0.002* |  |  |  |
| Health |  |  |  |  |  |  | 1.75 | [1.02, 2.47] | <0.001* |
| Observations |  | | |  | | |  | | |
| R^2^ / R^2^ adjusted | 0.053/0.046 | | | 0.070/0.062 | | | 0.151/0.144 | | |
| BIC | 1119 |  |  | 1117 |  |  | 1105 |  |  |
|  | **Model 2** | | | **Model 1** | | | **Model 1** | | |
|  | **EHQ.TOTAL** | | | **EHQ.TOTAL** | | | **EHQ.TOTAL** | | |
|  | *Estimates* | *95% CI* | *p* | *Estimates* | *95% CI* | *p* | *Estimates* | *95% CI* | *p* |
| (Intercept) | 63.92 | [58.22, 69.62] | <0.001* | 58.57 | [53.25, 63.88] | <0.001* | 68.9 | [63.08, 74.73] | <0.001* |
| Natural Concerns | 1.21 | [0.70, 1.72] | <0.001* |  |  |  |  |  |  |
| Weight Control |  |  |  | 1.85 | [1.35, 2.36] | <0.001* |  |  |  |
| Social Image |  |  |  |  |  |  | 1.29 | [0.35, 2.23] | 0.008* |
| Observations |  | | |  | | |  | | |
| R^2^ / R^2^ adjusted | 0.221/0.203 | | | 0.291/0.286 | | | 0.054/0.047 | | |
| BIC | 1103 |  |  | 1081 |  |  | 1119 |  |  |

Abbreviations: EHQ, Eating Habits Questionnaire; CI, Confidence Interval; BIC, Bayesian Information Criterion

Statistical analysis completed via bivariate linear regression

*Statistical significance, *p*<0.05

**Table S3**: Relationship between Compulsive Exercise (CET) and orthorexia (SCOFF ≥2) using binomial logistical regression with modelling

|  |  | **Model 1** | | | | **Model 2** | | | | **Model 3** | | | |
| --- | --- | --- | --- | --- | --- | --- | --- | --- | --- | --- | --- | --- | --- |
|  | **Model Parameters** | **Est.** | ***OR*** | ***95% CI*** | ***p*-value** | **Est.** | **OR** | ***95% CI*** | ***p*-value** | **Est.** | **OR** | ***95% CI*** | ***p*-value** |
| **CET** | (Intercept) | 4.55 | 94.54 | [15.77, 566.95] | <0.001* | 4.74 | 114.69 | [17.68, 744.00] | <0.001* | 8.10 | 3277.32 | [50.75, 211635.41] | <0.001* |
|  | CET | -0.33 | 0.72 | [0.63, 0.83] | <0.001* | -0.32 | 0.73 | [0.63, 0.84] | <0.001* | -0.60 | 0.55 | [0.40, 0.77] | <0.001* |
|  | COHORT |  |  |  |  |  |  |  |  |  |  |  |  |
|  | Athlete vs. Control |  |  |  |  | 0.01 | 1.01 | [0.42, 2.47] | 0.975 | 0.35 | 1.42 | [0.96, 2.10] | 0.076 |
|  | ED vs. Control |  |  |  |  | -1.98 | 0.14 | [0.03, 0.59] | 0.007* | 0.46 | 1.58 | [1.02, 2.44] | 0.039* |
|  | Athlete vs. ED |  |  |  |  | 2.00 | 7.34 | [1.78, 30.66] | 0.007* | -0.46 | 0.63 | [0.41, 0.98] | 0.039* |
|  | McFadden's R^2^/BIC | 0.16/155 | | |  | 0.21/155 | | |  | 0.24/160 | |  |  |

Abbreviations: SCCOF, ‘Sick, Control, One-Stone, Fat, Food’; Est, Estimate; OR, Odds Ratio, CI, Confidence Interval’ CET, Compulsive Exercise Test; ED, Eating Disorder; BIC, Bayesian Information Criterion

Statistical analysis conducted by sequential logistical regression, *statistically significant result, p<0.05

**Table S4:** Relationship between Compulsive Exercise Test (CET) and orthorexia nervosa (EHQ), using binomial logistical regression with modelling

|  | **Model 1** | | | **Model 2** | | | **Model 3** | | |
| --- | --- | --- | --- | --- | --- | --- | --- | --- | --- |
|  | **EHQ.TOTAL** | | | **EHQ.TOTAL** | | | **EHQ.TOTAL** | | |
| *Parameters* | *Estimates* | *95% CI* | *p* | *Estimates* | *95% CI* | *p* | *Estimates* | *95% CI* | *p* |
| (Intercept) | 47.43 | [37.6, 57.52] | <0.001* | 48.08 | [38.09, 58.08] | <0.001* | 47.49 | [32.24, 62.74] | <0.001* |
| CET | 2.32 | [1.52, 3.12] | <0.001* | 2.16 | [1.33, 2.98] | <0.001* | 2.21 | [0.89, 3.53] | 0.001 |
| Athlete vs. Control |  |  |  | 0.40 | [-5.35, 6.15] | 0.892 | 0.05 | [-15.67, 41.87] | 0.369 |
| ED vs. Control |  |  |  | 9.12 | [0.74, 17.51] | 0.033* | -0.30 | [-2.47, 1.86] | 0.783 |
| Athlete vs. ED |  |  |  | -8.72 | [-17.51, -0.74] | 0.038* | 0.35 | [-1.85, 2.55] | 0.752 |
| Observations |  | | |  | | |  | | |
| R^2^ / R^2^ adjusted | 0.453/0.205 | | | 0.486/0.236 | | | 0.487/0.237 | | |
| BIC | 1088 |  |  | 1092 |  |  | 1102 |  |  |

Abbreviations: EHQ, Eating Habits Questionnaire; CI, Confidence Interval; BIC, Bayesian Information Criterion

Statistical analysis completed via bivariate linear regression, *Statistical significance, *p*<0.05

Participant observes study advertisement and begins the online survey providing implied consent

Advertisement

Participant identifies as having a history of an eating disorder. They provide email address and consent to be contacted by researchers. Participants are unable to continue with survey at this time.

Completes eligibility screening questions

Does not meet eligibility for ED group. Excluded from study

Participant completes EDA-5 screening interview with APD

Participant does not have a history of an eating disorder and continues to complete the online survey

Screening

Participants with EDA-5 diagnosis of ‘severe’ provided with recommendations for psychological support services and excluded from study

Participant meets EDA-5 criteria of ‘mild’ or ‘moderate’ provided link to complete survey

Does not meet eligibility for ED group. Meets eligibility criteria for alternate cohort. Provided with link to complete survey

Participant meets eligibility criteria and is allocated to one of the following cohorts:

- Control
- Athlete
- Athlete

Eligibility

Participant provides letter from healthcare professional confirming ED diagnosis

Participant meets eligibility criteria for ED cohort

**Figure S1:** Recruitment process flow chart
